# Distinct Effects of Seed Coat and Flower Colors on Metabolite Contents and Antioxidant Activities in Safflower Seeds

**DOI:** 10.3390/antiox12040961

**Published:** 2023-04-19

**Authors:** Weilan Li, Eunae Yoo, Jungsook Sung, Sookyeong Lee, Sojeong Hwang, Gi-An Lee

**Affiliations:** National Agrobiodiversity Center, National Institute of Agricultural Sciences, Rural Development Administration, Jeonju 54874, Republic of Korea

**Keywords:** antioxidant activity, *Carthamus tinctorius* L., fatty acid, flower color, oil, phytochemical, seed coat color

## Abstract

Safflower is an important oilseed crop cultivated primarily for its seeds, which have pharmaceutical properties. Color is an important agronomical trait that appears to be a prior parameter for evaluating the internal quality of plant seeds. This study employs 197 safflower accessions seeds to analyze how their seed coat and flower colors affect their total oil content, fatty acid composition, total phenolic content (TPC), *N*-(*p*-coumaroyl)serotonin (CS) and *N*-feruloylserotonin (FS) contents, and [2,2-diphenyl-1-picrylhydrazyl (DPPH) and 2,2′-azino-bis-(3-ethylbenzothiazoline-6-sulfonic acid) (ABTS)] radical scavenging activities. Significant variations were observed in the targeted metabolite contents and antioxidant properties among genotypes. Notably, the linoleic acid content, total unsaturated fatty acid content, the ratio of total unsaturated fatty acid to total saturated fatty acid, CS, FS, ABTS, and DPPH scavenging capacities varied significantly based on seed coat color, with white-seeded genotypes having the highest average values of these parameters. Moreover, the linoleic acid content differed significantly (*p* < 0.05) among the genotypes with varying flower colors, with white-flowered accessions having the highest average content. Furthermore, genotypes K185105 (No. 75) and K175278 (No. 146) were identified as promising genetic resources with health benefits. Overall, these findings reveal that seed coat and flower colors distinctly affect metabolite contents and antioxidant properties in safflower seeds.

## 1. Introduction

Safflower (*Carthamus tinctorius* L.) is an annual oilseed crop belonging to the family *Asteraceae* or *Compositae*. It thrives under arid and cool agricultural conditions, making it an ideal plant for cultivation in China, India, the United States, Australia, Ethiopia, Europe, and Mexico, where it has been extensively grown [1,2,3,4]. Variations in plant height, branch number, head number and diameter, flower color, and seed coat color, as documented in several studies [4,5,6,7,8,9], indicate that the morphological characteristics of safflower are remarkably diverse. Safflower florets and seed oil have been used as condiment oils, colorants, cosmetics, and medicines, as well as for salad dressing and margarine production [10,11,12,13,14]. In particular, safflower seeds have garnered significant interest due to the presence of biologically active compounds that offer various health benefits.

Safflower seeds contain 35–50% oil, which comprises palmitic acid (4.2–12.0%), stearic acid (1.5–5.5%), oleic acid (9.2–83.4%), linoleic acid (10.5–82.6%), and a trace amount of linolenic acid [15,16,17,18]. Safflower seed oil has numerous health benefits for humans because it contains a high proportion of unsaturated fatty acids. For example, the functional activities of monounsaturated fatty acid (oleic acid) include decreasing the risk of cardiovascular disease by lowering systolic blood pressure; consumers favor this acid due to its high stability and bland flavor [16,19,20]. The main polyunsaturated fatty acids, linoleic and linolenic acids, play a role in controlling acne, reducing blood cholesterol levels, preventing coronary heart disease and myocardial infarction, and functioning as antiarrhythmic agents [21,22,23,24]. Notably, safflower seeds contain polyphenolic compounds and pigments that exhibit strong antioxidant properties, thereby indicating significant potential for use in pharmaceutical applications. Polyphenolic compounds possessing potent antioxidant properties represent a class of plant-based medicines that have been extensively studied for their efficacy in treating various diseases, including inflammation, dementia, and atherosclerosis [25,26,27]. *N*-(*p*-coumaroyl) serotonin (CS) and *N*-feruloylserotonin (FS) are the primary serotonin derivatives found in safflower seed extracts and are identified as phenolic constituents with biological effects [28]. As dietary antioxidants, CS and FS play important roles in preventing low-density lipoprotein accumulation, oxidation, and atherosclerosis [28,29].

Color is an important morphological trait that can provide valuable insights into the internal quality of plant seeds [30,31,32]. Pigments, as essential secondary metabolites, play multiple roles in plant development, flower and seed coloration, and photosynthesis. They exhibit potent biological activities and are used as medicinal agents to prevent and treat various diseases [30,33]. The safflower corolla exhibits varying colors, including red, yellow, white, orange, and cream [6]. Notably, the color characteristics and biological properties of safflower florets are correlated [31,33]. As a seed-growing organ tissue, the floral organ significantly contributes to the development of safflower seeds. However, studies exploring the relationship between floret color and biologically active compounds in safflower seeds remain unavailable. The seed coat color of safflower can be white, cream, brown, or black, among other colors [7,8,9], and it appears to be a prior parameter for evaluating seed quality and pharmaceutical values in terms of the seeds’ ability to produce secondary metabolites, which have various beneficial effects on human health [8,34]. Although several studies have explored the effect of seed coat color on the metabolites and antioxidative activities of safflower seeds [8,32], insights into this association remain limited, owing to the small number of genotypes included in these studies. To effectively predict the internal quality information of safflower seeds, the effects of flower and seed colors on their metabolite contents and antioxidant properties must be investigated using a large number of accessions.

In this study, 197 safflower germplasm accessions with three flower color types (red, yellow, and white) and five seed coat color types (white, mixed, light brown, brown, and dark brown) were cultivated under similar field conditions. Then, their total oil content, phytochemical contents, fatty acid profile, and antioxidant activities were investigated. Next, variations in total oil content, CS and FS content, total phenolic content (TPC), fatty acid profile, and DPPH and ABTS scavenging capacities among genotypes were examined. Multivariate analysis was used to analyze the effects of seed coat and flower colors on the total oil content, phytochemical contents, fatty acid profile, and antioxidant capacities of safflower seeds. Overall, this study expands the current understanding of safflower regarding the relationship between morphological traits and internal quality information, providing useful data for evaluating seed quality and developing superior safflower varieties.

## 2. Materials and Methods

### 2.1. Reagents and Chemicals

Folin-Ciocalteu phenol reagent, 2,2-diphenyl-1-picrylhydrazyl (DPPH), 2,2′-azino-bis-(3-ethylbenzothiazoline-6-sulfonic acid) (ABTS), ascorbic acid (AA), 6-hydroxy-2,5,7,8-tetramethylchroman-2-carboxylic acid (Trolox), potassium persulfate, 14% boron trifluoride-methanol (BF3-methanol), anhydrous sodium sulfate, *n*-hexane, sodium hydroxide, fatty acid standards (palmitic, stearic, oleic, linoleic, and linolenic acids), chloroform, ethanol, and methanol were purchased from Sigma Aldrich (St. Louis, MO, USA). CS and FS were purchased from Santa Cruz Biotechnology (Santa Cruz, CA, USA). All chemicals were of analytical grade and used without further purification.

### 2.2. Safflower Cultivation, Inspections, and Sample Preparation

The seeds of 197 safflower accessions were obtained from the gene bank of the National Agrobiodiversity Center, Rural Development Administration, Jeonju, Republic of Korea. All safflower genotypes were cultivated at the Herb Experiment Station, Medicinal Herb Resource Research Institute, Jeollabuk-Do Agricultural Research & Extension Services, Namwon (35°24′48.9″ N, 127°31′39.1″ E), in 2019. A space of 15 cm was made between the plants. Field and laboratory inspections were conducted to determine the morphological characteristics of the flowers and seed coat colors; the seeds were harvested at full maturity and dried in a VS-1202D drying oven (Vision Scientific, Bucheon, Republic of Korea) at 45 °C for 3 days. The dry seeds were crushed into a fine powder, sieved using a 315 µm screen, and then stored at −20 °C for further analysis.

### 2.3. Seed Crude Extract Preparation

Initially, crude extracts were prepared according to a previously reported method with some modifications [35,36]. The pulverized seeds (7 g) were mixed with 75% ethanol (20 mL) and then loaded onto an accelerated solvent extractor (ASE-350; Dionex, Sunnyvale, CA, USA). The extractions were conducted under nitrogen gas treatment, with the pressure and temperature adjusted to 1200 psi and 70 °C, respectively. The extract solution was transferred to a new tube and concentrated in a vacuum concentrator (HT-6; Genevac, Ipswich, UK) at 40 °C for 10 h. The concentrated extracts were re-dissolved in 75% ethanol and purified using a filter (0.45 µm) before analysis. The final concentrations for analysis were adjusted to 0.1 mg/mL for TPC, 0.2 mg/mL for antioxidant capacity assay, and 5 mg/mL for serotonin derivatives. Each sample was prepared in biological triplicate.

### 2.4. Determination of TPC

TPC was evaluated using the Folin-Ciocalteu colorimetric method [37], with some modification by Assefa et al. [35]. Each sample and standard (100 µL) were mixed with the Folin–Ciocalteu reagent (100 µL) and incubated at room temperature (20–25 °C) for 3 min. Next, the mixture was supplemented with 100 µL of 2% Na_2_CO_3_ and incubated in the dark at room temperature for 30 min. The reacted solutions were then subjected to absorbance measurement at 750 nm using an Eon Microplate Spectrophotometer (Bio-Tek, Inc., Winooski, VT, USA). Gallic acid was used as a standard for TPC quantification. Each sample was repeated three times, and the results were presented as µg gallic acid equivalent per mg sample (µg·GAE/mg).

### 2.5. Determination of Serotonin Derivative Contents

CS and FS concentrations were evaluated using ultra-high performance liquid chromatography [38]. Pure CS and FS were used as standards for identification and quantification. For the analysis, a solvent comprising A (0.1% formic acid in water) and B (0.1% formic acid in acetonitrile) was used as the mobile phase. The running was conducted according to the following gradient elution scheme: 0–6 min, 85% A and 15% B; 6–15 min, 85–60% A and 15–40% B; post-running for 5 min with 60–20% A and 40–80% B. The serotonin derivatives were detected at 324 nm using a C18 column (1.8 μm, 2.1 × 50 mm). The injection volume, flow rate, and temperature of the column were set for 2 µL, 0.4 mL/min, and 25 °C, respectively. The CS and FS contents were determined based on standard curves prepared from the standard solutions.

### 2.6. Antioxidant Capacity Assay

Antioxidant properties were evaluated using DPPH and ABTS assays. The DPPH radical scavenging activity was estimated based on the methodology described previously [39]. Briefly, 100 µL of purified crude extract and 150 µL of anhydrous ethanol (150 μM) were mixed by shaking vigorously, followed by 30-min incubation at 25 °C in the dark. By measuring the absorbance at 517 nm, the mixtures were used to determine the DPPH radical scavenging activity. The ABTS radical scavenging activity was evaluated following the method described by Re et al., with some modifications [40]. Briefly, the ABTS radical cation was produced by mixing ABTS stock solution (7 mM) and potassium persulfate (2.45 mM) in equal volumes, and incubated overnight at room temperature in the dark. Next, the mixture was diluted with methanol to obtain the ABTS radical cation solution, which had an absorbance of 0.7 ± 0.02 at 734 nm. Then, 10 μL of the sample or standard solution was mixed with 190 μL of the ABTS radical cation solution, followed by 6-min incubation at room temperature. The mixtures were used to evaluate the ABTS radical scavenging activity by measuring the absorbance at 734 nm. A spectrophotometer (Bio-Tek, Winooski, VT, USA) was used for all absorbance measurements.

The DPPH and ABTS radical scavenging activities were calculated using the following equation:

Antioxidant activity (%) = [1 − (Asample − Asample blank)/(Acontrol − Acontrol blank)] × 100,

where A represents absorbance. Ascorbic acid and Trolox were used as standards. DPPH and ABTS radical scavenging activities were expressed as mg ascorbic acid equivalent per g of dried seed weight (mg AAE/g) and µg Trolox equivalent per mg (µg·TE/mg) of the dried weight, respectively.

### 2.7. Determination of Total Oil Content

The total oil content was assessed following a previously described method with some modifications [17]. A Soxhlet apparatus (SoxtecTM 2043 system; OSS Tecator AB, Hillerod, Denmark) was applied for the extraction. One gram of dry pulverized seeds was mixed with 50 mL of *n*-hexane, followed by 30-min boiling, 60-min rinsing, and 30-min recovery at 135 °C. The weight of the obtained oil was measured after cooling at room temperature. The total oil content was calculated as a percentage (weight of obtained oil to the weight of seed sample used for the extraction). The experiments were conducted in triplicate.

### 2.8. Analysis of Fatty Acids

The percentages of fatty acids were determined as reported previously, with some modifications [17]. The fatty acid methyl esters were generated from crude fat by transmethylation. First, the oil extracts were mixed with 2 mL of 0.5 M NaOH, vortexed for 5 s, incubated in a water bath at 80 °C for 10 min, and finally cooled to room temperature. Next, 2 mL of 14% cold boron trifluoride-methanol was added to the mixture, vortexed for 5 s, re-incubated in a water bath at 80 °C for 10 min, and cooled to room temperature. Finally, the mixture was supplemented with 7 mL of *n*-hexane and 2 mL of H_2_O, vortexed for 10 s, and centrifuged (4 °C, 3000 rpm) for 10 min. The upper supernatant was collected and filtered through filter paper containing anhydrous sodium sulfate powder, and the resulting filtrate was stored at −20 °C in gas chromatography vials before analysis.

The fatty acid methyl esters were analyzed using the GCMS-QP2010 ultra gas chromatography instrument (Shimadzu Co., Kyoto, Japan) equipped with a HP-INNOWAX column (0.25 mm × 30 m, 0.25 μm). For the analysis, the column was initially set at 100 °C, and the running was conducted as follows: increase from 100 to 170 °C with a rate of 60 °C/min, holding for 1 min; increase from 170 to 240 °C with a rate of 60 °C/min, holding for 1 min. The temperature of both the injector and detector was set at 250 °C. The injection volume was 1 µL with a split ratio of 50, and helium (He) was used as a carrier gas with a flow rate of 1.5 mL/min. The fatty acid content was calculated and expressed as the percentage of total fatty acid using the peak areas.

### 2.9. Statistical Analysis

The experiments were conducted in triplicate. Results of total oil content, fatty acid profile, CS and FS content, TPC, and DPPH and ABTS values were recorded as mean ± standard deviation (SD). The data were subjected to multivariate statistical analysis, including analysis of variance, Pearson correlation, principal component analysis (PCA), and hierarchical clustering principal component (HCPC) analysis using R software (version 4.2.2; RStudio, Boston, MA, USA).

## 3. Results and Discussion

### 3.1. Morphological Traits

Figure S1 shows the color of the safflower petals and seeds after inspection. The flower color was inspected immediately after blooming, and three types of corolla color were recorded: red (*n* = 41), yellow (*n* = 153), and white (*n* = 3). The seed coat color was examined and classified into five groups: light brown (*n* = 89), brown (*n* = 16), dark brown (*n* = 3), white (*n* = 41), and mixed (*n* = 48). In addition, the genotypes with a mixed seed coat color showed that the seeds displayed different colored surfaces, or a white surface blended with other colors (light brown, brown, and dark brown) in varying degrees of complexity.

### 3.2. Diversity of Target Metabolite Content and Antioxidant Properties of 197 Safflower Accessions

Table 1 lists the wide variations observed in total oil content, fatty acid profile, TPC, CS and FS contents, and DPPH and ABTS values. The total oil content ranged from 10.15 to 38.37%, with a mean of 20.54%, corroborating previous findings in which safflower seed oil content ranged from 20 to 45% [16], from 22.10 to 38.77% [41], from 15.30 to 34.00% [42], and from 23.08 to 36.51% [43]. The main fatty acids were linoleic acid (65.66–84.19%) and oleic acid (8.09–25.55%), which comprised approximately 90% of the total fatty acid. The remaining 10% comprised palmitic acid (5.13–8.38%), stearic acid (1.87–5.31%), and a trace amount of linolenic acid (0.00–0.21%). Moreover, the ratio of total unsaturated fatty acid to total saturated fatty acid (US) was 8.01–12.86. The fatty acid profile observed in the present study showed wide variability and differed slightly from previous publications [42,43,44,45]. The CS and FS contents among the samples ranged from 0.20 to 62.01 mg/g and from 0.04 to 42.80 mg/g, respectively. TPC was measured between 21.95 and 147.13 µg·GAE/mg·DE, with a mean of 72.96 µg·GAE/mg·DE. In another study, Jung et al. documented similar values of CS (2.56–64.99 mg/g) and FS (1.92–65.36 mg/g), but a smaller range of TPC (28.25–90.53 µg·GAE/mg·DE), based on the analysis of 43 safflower genotypes [38]. Nevertheless, Assefa et al. reported a similar level of TPC (23.71–132.72 µg·GAE/mg·DE) among 237 safflower genotypes [17]. The ABTS and DPPH radical scavenging activities ranged from 23.59 to 304.38 µg·TE/mg·DE and 193.40 to 888.30 µg·AAE/mg·DE, respectively. In contrast to prior investigations, the antioxidant activity of ABTS and DPPH values showed a broader range of variability [18,38]. Among the variables evaluated in the present study, CS showed the highest coefficient of variation (67.45%), indicating a high level of genetic diversity among the safflower genotypes, whereas linoleic acid exhibited the lowest coefficient of variation (2.82%).

### 3.3. Association of Metabolite Content and Antioxidant Activities with Seed Coat Color

**Figure 1:**
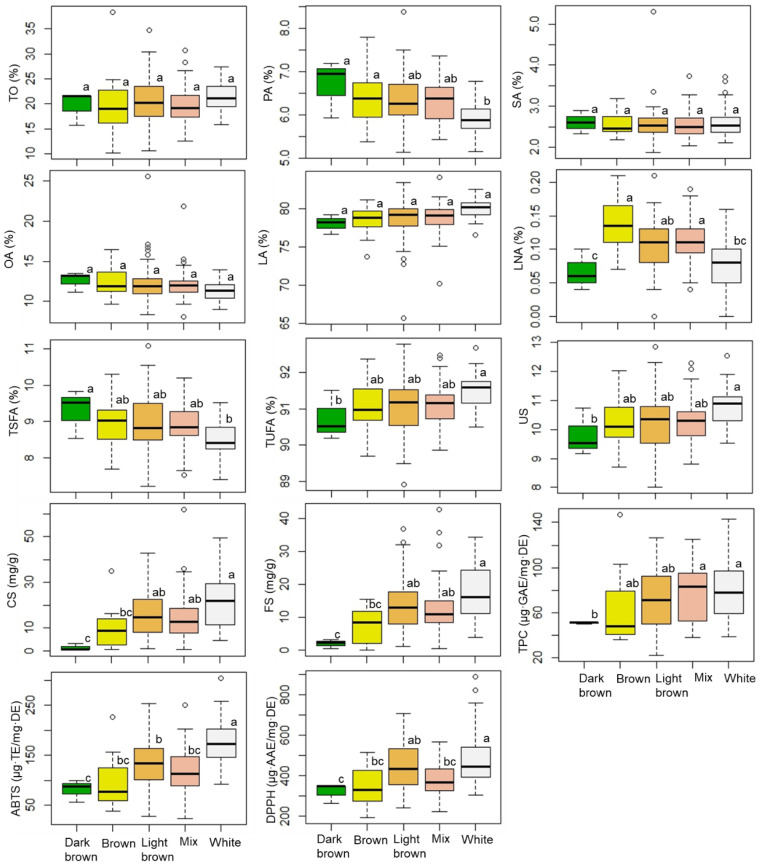
Variations in total oil content, fatty acid profile, phytochemical contents, and antioxidant activities of safflower seeds according to seed coat color. TO, total oil; PA, palmitic acid; SA, stearic acid; OA, oleic acid; LA, linoleic acid; LNA, linolenic acid; TSFA, total saturated fatty acid; TUFA, total unsaturated fatty acid; US, the ratio of TUFA to TSFA; CS, *N*-(*p*-coumaroyl) serotonin; FS, *N*-feruloylserotonin; TPC, total phenolic content; ABTS, ABTS radical scavenging activity; DPPH, DPPH radical scavenging activity. Values in the same row marked with different superscript letters are significantly different (*p* < 0.05).

Figure 1 and Table S1 present the results of the effects of seed coat color on the total oil content, fatty acid profile, TPC, CS and FS contents, and antioxidant activities of ABTS and DPPH. In accordance with prior investigations, the primary fatty acid in each seed coat color was linoleic acid, followed by oleic acid [8,15,16,42]. In addition, the fatty acid profile, serotonin derivatives, and antioxidant activities, with the exception of total oil content, stearic acid, oleic acid, and TPC, differed significantly (*p* < 0.05, 0.01, or 0.001) among accessions with different seed coat colors. Because genotypes with a varied seed coat color exhibited a colorful surface in the complexity index, which contributed to variations in metabolite content and antioxidant activity in their seeds, all the parameters observed in accessions with a mixed seed coat color showed moderate values between those with white and dark brown seed coat colors. Interestingly, the average values of total oil content, linoleic acid percentage, total unsaturated fatty acid content, US index, CS and FS contents, TPC, ABTS radical, and DPPH radical scavenging activities all decreased in an order of white > light brown > brown > dark brown seeds, whereas the average content of oleic acid, palmitic acid, and total saturated fatty acid increased in the order of white < light brown < brown < dark brown seeds. Consistent with other studies, genotypes with a white seed coat color exhibited higher levels of linoleic acid content than other seed-pigmented genotypes [8]. The higher amount of unsaturated fatty acids, phytochemical contents, and antioxidant activities indicated good quality and health benefits of the white seeds. Seed coat color is important for seed appearance and can affect the phytochemical contents and antioxidant properties of seeds of many crops, such as sesame [46], soybean [47], rice [48], sorghum [49], and safflower [8,32]. Accordingly, conducting research on the correlations between seed coat color, metabolite content, and antioxidant activities could contribute significantly to the selection of seeds with high quality and medicinal values. However, only a few studies have explored such associations in safflower. Karami et al. (2018) revealed that the black seed coat extracts exhibited stronger antioxidant activity than the white/brown seed coat extract; however, the black seed coat contained less total flavonoid than the white and other pigmented seed coats [8]. Tayebeh et al. (2021) evaluated the phytochemical contents and antioxidant properties in the seed coat extracts of genotype A82 (black seed) and genotype C111 (white seed) and found that the total flavonoid content and antioxidant activity did not differ significantly between the two genotypes [32]. Although these findings significantly contributed to safflower research, only a few genotypes were used, and these studies primarily concentrated on seed coat extracts. Therefore, a comprehensive study based on a large number of safflower genotypes is required to gain a thorough understanding of the interaction effect of seed coat color on the metabolite content and antioxidant properties of safflower seeds. Here, multivariate analysis performed on the metabolite content and antioxidant properties of the seeds of 197 safflower genotypes revealed that seed coat color could affect the metabolite content and antioxidant properties of the safflower seeds. In particular, accessions with a white seed coat color appeared to be excellent genetic resources with high levels of biologically active compound content and antioxidant capacity.

### 3.4. Association of Metabolite Content and Antioxidant Properties with Flower Color

The effects of flower color on the metabolite content and antioxidant properties of safflower seeds were also studied (Table 2). Similar to other studies [8,17,42], linoleic acid was the main fatty acid in each floral color. Among the three floral color types, only the fatty acid profiles of oleic acid (*p* < 0.05), linoleic acid (*p* < 0.05), and linolenic acid (*p* < 0.01) differed significantly. Furthermore, the white-flowered genotypes had the highest average values for linoleic acid content (81.93%), linolenic acid content (0.14%), total unsaturated fatty acid content (91.58%), US index (10.96), and TPC (80.46 µg·GAE/mg·DE) compared to the red/yellow-flowered genotypes, indicating that white-flowered genotypes could be important resources owing to their high amount of unsaturated fatty acids in the seeds. In addition, the average values of total oil content, stearic acid percentage, and antioxidant activities of ABTS and DPPH decreased in an order of yellow > red > white-flowered genotypes, whereas the average values of stearic acid content and TPC displayed an inverse trend. Despite these differences, flower color did not result in significant differences (*p* < 0.05) in the values of these parameters. Several studies have shown that the metabolic profiles of safflower florets are associated with flower color, which plays an important role in their biological activities [31,50,51,52]. Notably, the main component of the safflower floral organ is the seeds contained inside, and the floral organ is crucial for the development of safflower seeds. However, the association between flower color and metabolic profiles of safflower seeds has not been adequately studied. This study analyzed 197 safflower accessions to determine the sensitivity of metabolic content and antioxidant properties to flower color and found that white-flowered accessions could be important resources, as their seeds displayed high levels of linoleic acid. However, only three accessions (K186514, K185103, and K185106) developed white flowers, and further studies of such genetic resources are highly recommended.

### 3.5. Pearson Correlation Analysis

Table 3 lists the results of the Pearson correlation analysis of total oil content, fatty acid profile, TPC, CS and FS contents, and DPPH and ABTS radical scavenging activities. Linoleic acid was inversely associated with oleic acid (r = −0.96; *p* < 0.001), corroborating the findings of other studies (r = −0.973; *p* < 0.01) [18], (r = −0.9996; *p* < 0.05) [17], and (r = −0.89; *p* < 0.01) [53]. The enzyme activity of FAD2 oleate Δ12 desaturase could clarify the mechanism underlying the negative relationship between oleic acid and linoleic acid [54]. The total oil content was significantly and positively associated with linoleic acid content (r = 0.41; *p* < 0.01), and ABTS ((r = 0.35; *p* < 0.01) and DPPH (r = 0.26; *p* < 0.01) radical scavenging activities. In addition, the CS and FS contents, TPC, and ABTS and DPPH radical scavenging activities were all significantly and positively correlated, corroborating previous findings [18,38]. These findings support the notion that secondary phytochemical contents are positively linked with the capacity to remove free radicals.

### 3.6. PCA and HCPC Results

**Figure 2:**
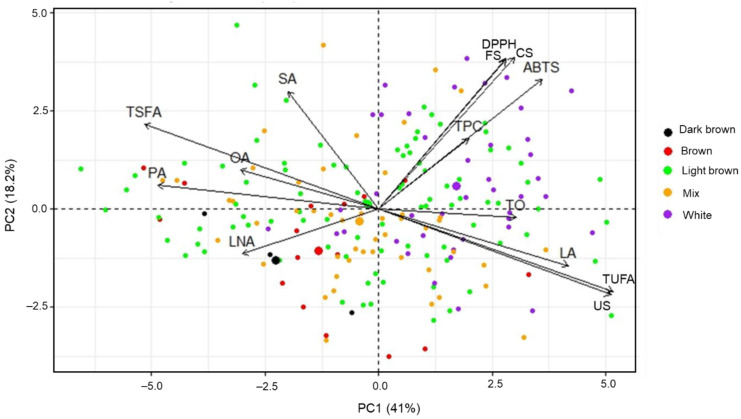
Principal component biplot for safflower seeds based on their total oil content, fatty acid profile, phytochemical contents, and antioxidant activities using the entire dataset. TO, total oil; PA, palmitic acid; SA, stearic acid; OA, oleic acid; LA, linoleic acid; LNA, linolenic acid; TSFA, total saturated fatty acid; TUFA, total unsaturated fatty acid; US, the ratio of TUFA to TSFA; CS, *N*-(*p*-coumaroyl)serotonin; FS, *N*-feruloylserotonin; TPC, total phenolic content; ABTS, ABTS radical scavenging activity; DPPH, DPPH radical scavenging activity.

The entire dataset of total oil content, fatty acid profile, TPC, CS and FS contents, and ABTS and DPPH radical scavenging activities was subjected to the multivariate modeling and chemometric techniques of PCA. Table S2 lists the parameters of the first five principal components (PCs) obtained from a set of 14. PCs 1–4 had eigenvalues greater than one and contributed 79.08% of the total variations (41.01, 18.17, 10.37, and 9.53%, respectively). The score plot and loading of variables generated by PC1 and PC2 were used to determine the distributions and correlations of the total oil content, TPC, CS and FS contents, fatty acid profile, ABTS and DPPH radical scavenging activities, and the safflower accessions (Figure 2). PC1 had high contributions from palmitic acid (12.18%), total saturated fatty acid (13.76%), total unsaturated fatty acid (13.75%), and US index variables, whereas PC2 was mainly contributed by stearic acid (10.46%), CS (17.38%), FS (16.89%), ABTS (12.65%), and DPPH (17.09%). The correlations displayed by PCA were determined by the angle between the variables, with an angle less than 90° indicating a positive association [55]. As shown in Figure 2, the correlations of variables visualized by PCA agreed well with the results obtained by Pearson correlation analysis and were similar to other studies [17,18,38,53]. Moreover, most genotypes with white seed coat colors were distributed along the positive side of PC1, while most accessions with brown and dark brown seed coat colors were on the negative side of PC1. In particular, distinctive aggregations between genetic resources with seed coat colors of white and dark brown, white and brown, white and mixed, and white and light brown were observed (Figure 2 and Figure S2). Similarly, Karami et al. (2018) used PCA to analyze the phytochemical content and antioxidant activity of safflower seed coat extract and observed distinct groups between accessions with black and white/brown seed coat color [8]. The distinctions between genotypes with different seed coat colors revealed wide variabilities in their seeds’ total oil content, fatty acid profile, TPC, CS and FS contents, and ABTS and DPPH radical scavenging activities. Our results suggest that safflower seed coat color could be used as an important appearance factor for assessing internal seed quality.

**Figure 3:**
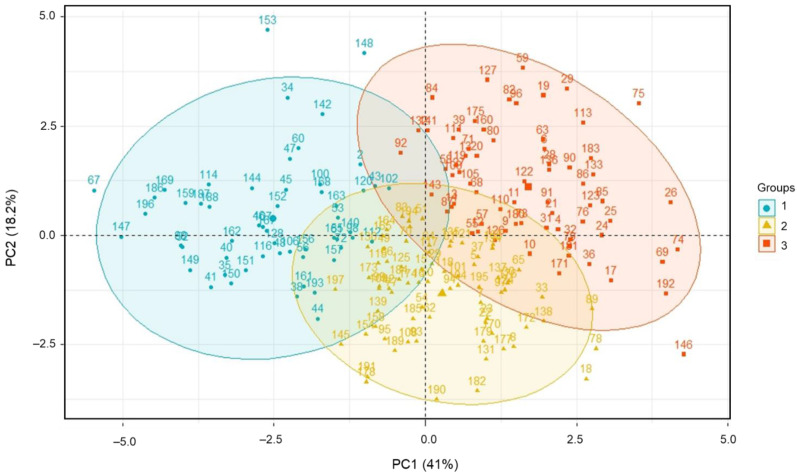
Hierarchical clustering principal component analysis biplot showing safflower genetic resource clustering based on total oil content, fatty acid profile, phytochemical contents, and antioxidant activities using the entire dataset.

HCPC analysis with the FactoMineR program [36,56] was used to classify genotypes based on the entire dataset of metabolite content and antioxidant properties. Figure 3 illustrates the scatter and loading plots, which were computed by hierarchical clustering on principal components. In all, 197 safflower genotypes were clustered into three groups, and the related parameters for each group are listed in Table S3. Table S4 lists the safflower accessions used in the present study. Groups I, II, and III contained 55, 73, and 69 clustered accessions, respectively. Group I had the highest levels of palmitic acid, stearic acid, oleic, linolenic acid, and total saturated fatty acid contents. Group III had the highest values of total oil content, linoleic acid, total unsaturated fatty acid, US index, CS, FS, TPC, and ABTS and DPPH radical scavenging activities. In addition, Group III was characterized by genotypes K185105 (No. 75) and K175278 (No. 146), which possessed significant antioxidant activities and unsaturated fatty acid content, respectively. Therefore, these two accessions hold great promise as potent genetic resources that could contribute to the pharmaceutical application and development of improved varieties with health-promoting effects.

## 4. Conclusions

This study analyzed the total oil content, CS and FS contents, TPC, fatty acid profile, and ABTS and DPPH radical scavenging activities of 197 safflower accessions seeds. The metabolite content and antioxidant properties of the examined genotypes were found to vary significantly. The seed coat and flower colors were significantly associated with the targeted metabolite content and antioxidant activities in the seeds. By comparison, the genotypes with white seed coat colors had higher levels of target metabolite content and antioxidant activities. Linoleic acid content varied significantly among the genotypes with different flower colors, with the white flower color indicating high linoleic acid content in the safflower seeds. Hence, seed coat color was suggested as a useful parameter for discriminating genetic resources with health benefits. Furthermore, the white flower color indicated a high percentage of linoleic acid in seeds. Genotypes K185105 (No. 75) and K175278 (No. 146) were identified as potentially valuable genetic resources, with high levels of antioxidant activities and unsaturated fatty acid content, respectively. These findings are significant for safflower research and the development of novel safflower varieties with potent bioactivities. Further studies will be needed to investigate other health-promoting nutrients and agriculture traits, to provide valuable information for the selection of the best plant materials and desired agriculture traits.

## Figures and Tables

**Table 1:** Summary statistics of total oil content, fatty acid profile, phytochemical contents, and antioxidant activities of 197 safflower accessions’ seeds.

Parameter	Maximum	Minimum	Mean	CV (%)
TO (%)	38.37	10.15	20.54	20.85
PA (%)	8.38	5.13	6.26	8.68
SA (%)	5.31	1.87	2.58	14.02
OA (%)	25.55	8.09	12.02	16.00
LA (%)	84.19	65.66	79.06	2.82
LNA (%)	0.21	0.00	0.10	42.79
TSFA (%)	11.09	7.21	8.83	7.38
TUFA (%)	92.79	88.91	91.18	0.72
US	12.86	8.01	10.38	8.03
CS (mg/g)	62.01	0.20	15.84	67.45
FS (mg/g)	42.80	0.04	13.46	61.05
TPC (µg·GAE/mg·DE)	147.13	21.95	72.96	34.13
ABTS (µg·TE/mg·DE)	304.38	23.59	135.28	37.94
DPPH (µg·AAE/mg·DE)	888.30	193.40	430.10	27.49

TO, total oil; PA, palmitic acid; SA, stearic acid; OA, oleic acid; LA, linoleic acid; LNA, linolenic acid; TSFA, total saturated fatty acid; TUFA, total unsaturated fatty acid; US, the ratio of TUFA to TSFA; CS, *N*-(*p*-coumaroyl) serotonin; FS, *N*-feruloylserotonin; TPC, total phenolic content; ABTS, ABTS radical scavenging activity; DPPH, DPPH radical scavenging activity; SD, standard deviation; CV, coefficient of variation.

**Table 2:** Variations in total oil content, fatty acid profile, phytochemical contents, and antioxidant activities according to flower color.

Parameter	Values	Red	White	Yellow	*p*-Value
TO (%)	Range	10.15–38.37	13.67–23.51	10.58–34.67	NS
	Mean	19.42 ^a^	19.12 ^a^	20.86 ^a^	
	CV (%)	24.82	26.20	19.61	
PA (%)	Range	5.49–7.49	5.42–6.39	5.13–8.38	‧
	Mean	6.42 ^a^	5.90 ^a^	6.22 ^a^	
	CV (%)	8.88	8.31	8.52	
SA (%)	Range	1.98–3.28	2.11–3.19	1.87–5.31	NS
	Mean	2.53 ^a^	2.52 ^a^	2.59 ^a^	
	CV (%)	10.67	23.41	14.67	
OA (%)	Range	10.08–17.12	8.09–10.82	8.35–25.55	*
	Mean	12.41 ^a^	9.52 ^b^	11.96 ^a^	
	CV (%)	13.13	14.39	16.47	
LA (%)	Range	72.72–81.71	80.42–84.19	65.66–83.47	*
	Mean	78.54 ^b^	81.93 ^a^	79.14 ^b^	
	CV (%)	2.69	2.44	2.81	
LNA (%)	Range	0.04–0.21	0.10–0.19	0.00–0.21	**
	Mean	0.12 ^ab^	0.14 ^a^	0.10 ^b^	
	CV (%)	29.17	35.71	40.00	
TSFA (%)	Range	7.65–10.19	7.53–9.08	7.21–11.09	NS
	Mean	8.94 ^a^	8.42 ^a^	8.81 ^a^	
	CV (%)	7.83	9.50	7.26	
TUFA (%)	Range	89.82–92.39	90.92–92.47	88.91–92.79	NS
	Mean	91.07 ^a^	91.58 ^a^	91.20 ^a^	
	CV (%)	0.77	0.87	0.70	
US	Range	8.81–12.07	10.01–12.28	8.01–12.86	NS
	Mean	10.25 ^a^	10.96 ^a^	10.41 ^a^	
	CV (%)	8.49	10.77	7.88	
CS (mg/g)	Range	1.41–40.18	5.92–11.62	0.20–62.01	NS
	Mean	15.97 ^a^	8.50 ^a^	15.94 ^a^	
	CV (%)	56.29	34.00	70.01	
FS (mg/g)	Range	1.38–33.62	8.57–11.55	0.04–42.80	NS
	Mean	13.79 ^a^	10.16 ^a^	13.44 ^a^	
	CV (%)	49.82	14.76	64.21	
TPC (µg·GAE/mg·DE)	Range	37.63–143.30	39.74–105.65	21.95–147.13	NS
	Mean	76.14 ^a^	80.46 ^a^	71.96 ^a^	
	CV (%)	34.82	44.23	33.84	
ABTS (µg·TE/mg·DE)	Range	38.38–250.99	80.69–143.86	23.59–304.38	NS
	Mean	133.86 ^a^	122.08 ^a^	135.92 ^a^	
	CV (%)	37.05	29.37	38.43	
DPPH (µg·AAE/mg·DE)	Range	265.23–703.93	276.40–348.82	193.43–888.29	NS
	Mean	421.26 ^ab^	312.25 ^b^	434.77 ^a^	
	CV (%)	21.30	11.60	28.70	

TO, total oil; PA, palmitic acid; SA, stearic acid; OA, oleic acid; LA, linoleic acid; LNA, linolenic acid; TSFA, total saturated fatty acid; TUFA, total unsaturated fatty acid; US, the ratio of TUFA to TSFA; CS, *N*-(*p*-coumaroyl) serotonin; FS, *N*-feruloylserotonin; TPC, total phenolic content; ABTS, ABTS radical scavenging activity; DPPH, DPPH radical scavenging activity. Values in the same row marked with different superscript letters are significantly different (*p* < 0.05). NS, **‧**, *, **represent no significant or significant at *p* < 0.1, 0.05, 0.01, respectively.

**Table 3:** Pearson correlation coefficients of total oil content, fatty acid profile, phytochemical content, and antioxidant activities.

	TO	PA	SA	OA	LA	LNA	TSFA	TUFA	US	CS	FS	TPC	ABTS
PA	−0.32 ***												
SA	−0.18 **	0.00											
OA	−0.35 ***	0.32 ***	0.11										
LA	0.41 ***	−0.53 ***	−0.25 ***	−0.96 ***									
LNA	−0.37 ***	0.55 ***	−0.18 *	0.13	−0.24 ***								
TSFA	−0.37 ***	0.83 ***	0.55 ***	0.33 ***	−0.58 ***	0.36 ***							
TUFA	0.37 ***	−0.84 ***	−0.54 ***	−0.32 ***	0.58 ***	−0.37 ***	−1.00 ***						
US	0.35 ***	−0.83 ***	−0.55 ***	−0.33 ***	0.58 ***	−0.34 ***	−0.99 ***	0.99 ***					
CS	0.11	−0.29 ***	−0.03	−0.14	0.20 **	−0.14 *	−0.25 ***	0.26 ***	0.24 ***				
FS	0.14	−0.25 ***	−0.03	−0.13	0.18 *	−0.10	−0.22 **	0.22 **	0.21 **	0.85 ***			
TPC	0.08	−0.25 ***	0.03	−0.11	0.16 *	−0.17 *	−0.19 **	0.19 **	0.20 **	0.36 ***	0.33 ***		
ABTS	0.35 ***	−0.40 ***	0.04	−0.26 ***	0.32 ***	−0.47 ***	−0.31 ***	0.32 ***	0.31 ***	0.58 ***	0.54 ***	0.33 ***	
DPPH	0.26 ***	−0.30 ***	0.10	−0.09	0.14 *	−0.42 ***	−0.20 **	0.20 **	0.19 **	0.60 ***	0.56 ***	0.14 *	0.67 ***

TO, total oil; PA, palmitic acid; SA, stearic acid; OA, oleic acid; LA, linoleic acid; LNA, linolenic acid; TSFA, total saturated fatty acid; TUFA, total unsaturated fatty acid; US, the ratio of TUFA to TSFA; CS, *N*-(*p*-coumaroyl) serotonin; FS, *N*-feruloylserotonin; TPC, total phenolic content; ABTS, ABTS radical scavenging activity; DPPH, DPPH radical scavenging activity. *, **, *** represent significant at *p* < 0.05, 0.01, 0.001, respectively.

## Data Availability

Data are provided in the article and Supplementary Materials.

## Supplementary Materials

The following supporting information can be downloaded at: https://www.mdpi.com/article/10.3390/antiox12040961/s1, Table S1: Variations in total oil content, fatty acid profile, phytochemical contents, and antioxidant activities according to seed coat color; Table S2: Principal component analysis of total oil content, fatty acid profile, phytochemical contents, and antioxidant activities of 197 safflower accessions, with eigenvalues and individual and cumulative contributions of variables in the first five principal components; Table S3: Average cluster values of total oil content, fatty acid profile, phytochemical contents, and antioxidant activities of 197 safflower accessions; Table S4: Safflower genotypes used in this study; Figure S1: Flower and seed samples of safflower of different flower and seed coat colors; Figure S2: Principal component biplots of the total oil content, fatty acid profile, phytochemical contents, and antioxidant activities of the different seed coat color groups. (A) Loading plots of genotypes with white and brown seed coat colors. (B) Loading plots of genotypes with white and dark brown seed coat colors. (C) Loading plots of genotypes with white and mix seed coat colors. (D) Loading plots of genotypes with white and light green seed coat colors.
