# Supplementary material for: Distinct Effects of Seed Coat and Flower Colors on Metabolite Contents and Antioxidant Activities in Safflower Seeds
Source: Antioxidants (Basel). 2023 Apr 19;12(4):961. doi: 10.3390/antiox12040961 (PMC10136218; doi:10.3390/antiox12040961)
Supplement: Supplementary file 1 [file antioxidants-12-00961-s001.zip › antioxidants-2298144-supplementary.pdf]

Table S1. Variations in total oil content, fatty acid profile, phytochemical contents, and antioxidant activities according to seed coat color.

**Parameter**	**Values**	**Dark brown**	**Brown**	**Light brown**	**Mix**	**White**	***P*-Value**
TO (%)	Range	15.71–21.56	10.15–38.37	10.58–34.67	12.60–30.69	15.85–27.45	NS
	Mean	19.59[a]	19.79[a]	20.69[a]	19.70[a]	21.54[a]	
	CV (%)	17.15	31.83	22.14	19.70	13.42	
PA (%)	Range	5.93–7.19	5.38–7.79	5.13–8.38	5.42–7.35	5.15–6.77	***
	Mean	6.69[a]	6.41[a]	6.34[ab]	6.32[ab]	5.90[b]	
	CV (%)	10.01	9.98	9.15	7.59	4.75	
SA (%)	Range	2.32–2.89	2.19–3.19	1.87–5.31	2.04–3.74	2.11–3.71	NS
	Mean	2.60[a]	2.56[a]	2.57[a]	2.56[a]	2.62[a]	
	CV (%)	11.15	10.94	15.18	12.50	14.89	
OA (%)	Range	11.16–13.43	9.64–16.46	8.35–25.55	8.09–21.83	9.05–13.98	NS
	Mean	12.58[a]	12.34[a]	12.17[a]	12.16[a]	11.35[a]	
	CV (%)	9.86	13.70	18.24	15.87	9.60	
LA (%)	Range	76.70–79.27	73.72–81.17	65.66–83.47	70.23–84.19	76.63–82.56	*
	Mean	78.09[a]	78.56[a]	78.82[a]	78.87[a]	80.08[a]	
	CV (%)	1.66	2.48	3.26	2.70	1.56	
LNA (%)	Range	0.04–0.10	0.07–0.21	0.00–0.21	0.04–0.19	0.00–0.16	***
	Mean	0.07[c]	0.14[a]	0.10[ab]	0.11[a]	0.07[bc]	
	CV (%)	42.86	28.57	40.00	27.27	57.14	
TSFA (%)	Range	8.52–9.83	7.68–10.30	7.21–11.09	7.53–10.19	7.39–9.51	**
	Mean	9.29[a]	8.98[ab]	8.91[ab]	8.87[ab]	8.52[b]	
	CV (%)	7.32	8.02	7.86	6.65	5.63	
TUFA (%)	Range	90.19–91.51	89.70–92.37	88.91–92.79	89.85–92.47	90.49–92.68	**
	Mean	90.74[b]	91.03[ab]	91.09[ab]	91.14[ab]	91.5[a]	
	CV (%)	0.76	0.80	0.77	0.65	0.52	
US	Range	9.18–10.74	8.71–12.03	8.01–12.86	8.81–12.28	9.52–12.55	*
	Mean	9.81[b]	10.21[ab]	10.29[ab]	10.32[ab]	10.78[a]	
	CV (%)	8.36	8.91	8.55	7.46	6.12	
CS (mg/g)	Range	0.20–3.32	0.69–34.96	0.76–42.82	0.51–62.01	4.52–49.33	***
	Mean	1.41[c]	9.66[bc]	15.69[ab]	14.48[ab]	21.21[a]	
	CV (%)	119.15	89.44	60.74	74.45	54.13	
FS (mg/g)	Range	0.51–3.33	0.04–15.60	1.29–36.74	0.61–42.80	3.88–34.31	***
	Mean	2.05[c]	7.80[bc]	13.48[ab]	12.65[ab]	17.43[a]	
	CV (%)	69.76	64.62	56.16	65.69	49.57	
TPC (µg·GAE/mg·DE)	Range	49.73–51.69	35.93–147.13	21.95–126.28	37.64–124.87	38.43–143.30	·
	Mean	50.85[b]	62.44[ab]	71.4[ab]	75.85[a]	78.67[a]	
	CV (%)	1.99	50.54	34.43	31.71	29.16	
ABTS (µg·TE/mg·DE)	Range	56.55–100.20	38.38–226.36	28.53–253.44	23.59–250.99	92.45–304.38	***
	Mean	81.5[c]	94.05[bc]	134.29[b]	119.99[bc]	175.35[a]	
	CV (%)	27.58	54.31	35.42	35.71	25.14	
DPPH (µg·AAE/mg·DE)	Range	262.31–351.15	193.43–513.21	241.28–707.58	222.42–567.76	305.36–888.29	***
	Mean	320.82[c]	351.62[bc]	445.33[ab]	378.56[bc]	495.95[a]	
	CV (%)	15.80	26.98	25.06	21.17	27.43	

TO, total oil; PA, palmitic acid; SA, stearic acid; OA, oleic acid; LA, linoleic acid; LNA, linolenic acid; TSFA, total saturated fatty acid; TUFA, total unsaturated fatty acid; US, the ratio of TUFA to TSFA; CS, *N*-(*p*-coumaroyl) serotonin; FS, *N*-feruloylserotonin; TPC, total phenolic content; ABTS, ABTS radical scavenging activity; DPPH: DPPH radical scavenging activity. Values in the same row marked with different superscript letters are significantly different ($p < 0.05$). NS, ·, *, **, *** represent no significant or significant at $p < 0.1$, 0.05, 0.01, 0.001, respectively.

Table S2. Principal component analysis of total oil content, fatty acid profile, phytochemical contents, and antioxidant activities of 197 safflower accessions, with eigenvalues and individual and cumulative contributions of variables in the first five principal components.

**Variable**	**PC1**	**PC2**	**PC3**	**PC4**	**PC5**
TO	4.74	0.05	8.28	0.30	20.31
PA	12.18	0.42	0.04	9.56	5.98
SA	2.07	10.46	15.58	9.94	11.24
OA	4.78	1.13	28.95	18.61	0.73
LA	9.08	2.44	16.76	11.13	0.66
LNA	4.64	1.54	6.08	30.89	0.04
TSFA	13.76	5.41	4.04	0.69	0.03
TUFA	13.75	5.18	4.15	0.92	0.07
US	13.53	5.60	4.19	0.63	0.13
CS	4.63	17.38	5.18	6.87	0.00
FS	4.04	16.89	5.17	8.62	0.06
TPC	2.04	3.77	0.70	0.62	47.73
ABTS	6.71	12.65	0.88	0.19	1.71
DPPH	4.05	17.09	0.00	1.02	11.31
Eigenvalue	5.74	2.54	1.45	1.33	0.96
Variability (%)	41.01	18.17	10.37	9.53	6.86
Cumulative (%)	41.01	59.19	69.55	79.08	85.94

TO, total oil; PA, palmitic acid; SA, stearic acid; OA, oleic acid; LA, linoleic acid; LNA, linolenic acid; TSFA, total saturated fatty acid; TUFA, total unsaturated fatty acid; US, the ratio of TUFA to TSFA; CS, *N*-(*p*-coumaroyl) serotonin; FS, *N*-feruloylserotonin; TPC, total phenolic content; ABTS, ABTS radical scavenging activity; DPPH: DPPH radical scavenging activity.

Table S3. Average cluster values of total oil content, fatty acid profile, phytochemical contents, and antioxidant activities of 197 safflower accessions.

**Cluster**	**No. Acc.**	**TO (%)**	**PA (%)**	**SA (%)**	**OA (%)**	**LA (%)**	**LNA (%)**	**TSFA (%)**
I	55	17.47 ± 3.10[b]	6.88 ± 0.46[a]	2.73 ± 0.44[a]	13.40 ± 2.33[a]	76.87 ± 2.29[b]	0.13 ± 0.03[a]	9.61 ± 0.41[a]
II	73	21.33 ± 4.45[a]	6.07 ± 0.35[b]	2.49 ± 0.28[b]	11.63 ± 1.62[b]	79.73 ± 1.63[a]	0.10 ± 0.04[b]	8.56 ± 0.39[b]
III	69	22.14 ± 3.65[a]	5.96 ± 0.32[b]	2.55 ± 0.33[b]	11.33 ± 1.18[b]	80.10 ± 1.38[a]	0.08 ± 0.04[b]	8.50 ± 0.49[b]
P–value		***	***	**	***	***	***	***
	TUFA (%)	US	CS (mg/g)	FS (mg/g)	TPC (µg·GAE/mg·DE)	ABTS (µg·TE/mg·DE)	DPPH (µg·AAE/mg·DE)	
I	90.40 ± 0.42[b]	9.42 ± 0.44[b]	11.11 ± 6.87[b]	10.14 ± 6.34[b]	64.77 ± 25.26[b]	101.21 ± 38.89[c]	386.58 ± 92.00[b]	
II	91.45 ± 0.40[a]	10.71 ± 0.56[a]	9.49 ± 5.64[b]	8.67 ± 4.50[b]	66.35 ± 22.57[b]	117.39 ± 35.93[b]	365.76 ± 69.50[b]	
III	91.51 ± 0.49[a]	10.80 ± 0.70[a]	26.32 ± 9.22[a]	21.19 ± 6.88[a]	86.47 ± 21.32[a]	181.36 ± 39.86[a]	532.83 ± 108.43[a]	
P–value	***	***	***	***	***	***	***	

TO, total oil; PA, palmitic acid; SA, stearic acid; OA, oleic acid; LA, linoleic acid; LNA, linolenic acid; TSFA, total saturated fatty acid; TUFA, total unsaturated fatty acid; US, the ratio of TUFA to TSFA; CS, *N*-(*p*-coumaroyl) serotonin; FS, *N*-feruloylserotonin; TPC, total phenolic content; ABTS, ABTS radical scavenging activity; DPPH: DPPH radical scavenging activity. Values in the same row marked with different superscript letters are significantly different ($p < 0.05$). **, *** represent no significant or significant at $p < 0.01$, 0.001, respectively.

Table S4. Safflower genotypes used in this study.

No	Accession	No	Accession	No	Accession	No	Accession	No	Accession
1	IT333504	41	K185024	81	IT333472	121	IT333482	161	IT183710
2	K185768	42	IT333508	82	IT333486	122	IT333480	162	IT209543
3	K186709	43	K229389	83	K185025	123	IT333464	163	IT209509
4	K185765	44	K186563	84	IT333454	124	IT333460	164	IT202728
5	IT333456	45	K185227	85	K186562	125	IT333449	165	IT209523
6	K185113	46	K186730	86	K186564	126	IT333478	166	IT183707
7	IT333495	47	K229387	87	IT333473	127	IT333497	167	IT183706
8	IT333500	48	K185100	88	K185390	128	IT333485	168	909227
9	IT333475	49	K131657	89	K185418	129	K184502	169	909228
10	K185962	50	K185305	90	K185101	130	K184929	170	K184528
11	K229393	51	K185303	91	IT333489	131	IT333447	171	K131659
12	K185129	52	K185021	92	K131660	132	IT333448	172	K019148
13	K185719	53	K185772	93	IT333476	133	IT333450	173	K131656
14	K185109	54	K185054	94	K185103	134	K185222	174	IT333445
15	IT333490	55	IT333488	95	K185415	135	K185026	175	K184472
16	K185307	56	K185012	96	IT333474	136	IT333501	176	K184530
17	K185308	57	K185014	97	IT333458	137	K186565	177	K184536
18	K186514	58	K185230	98	K185803	138	K184505	178	K184529
19	IT333468	59	IT333511	99	K185106	139	K185782	179	K184535
20	IT333494	60	K185112	100	K229396	140	K185386	180	K184531
21	IT333505	61	K184983	101	K185770	141	IT333484	181	IT333459
22	K185228	62	K185142	102	K261449	142	K131662	182	K184524
23	IT333513	63	IT333492	103	IT333479	143	K171322	183	K014611
24	IT333506	64	IT333512	104	IT333446	144	IT333496	184	K184533
25	IT333467	65	IT333453	105	IT333457	145	K184480	185	K184520
26	IT333465	66	IT333455	106	K184931	146	K175278	186	K131653
27	K185414	67	K185229	107	K185016	147	K185017	187	K184478
28	IT333469	68	K186365	108	K185013	148	K185785	188	K184523
29	IT333498	69	IT333509	109	K185417	149	K186732	189	K184521
30	IT333481	70	K185771	110	IT333477	150	K185309	190	K184534
31	IT333503	71	K185311	111	IT333444	151	K185961	191	K184525
32	IT333487	72	IT333499	112	K171354	152	K185028	192	IT333491
33	K185058	73	IT333451	113	IT333452	153	K185784	193	K184526
34	K131655	74	K185015	114	K184966	154	K185099	194	K184527
35	K186729	75	IT333493	115	K184923	155	K185022	195	K171325
36	IT333462	76	K185107	116	K184922	156	K185104	196	K184484
37	IT333470	77	K185781	117	IT333510	157	K185224	197	K184477
38	IT333466	78	IT333471	118	IT333502	158	K185135		
39	IT333463	79	IT333461	119	K185046	159	K185232		
40	K186728	80	K184965	120	K229394	160	IT209562		

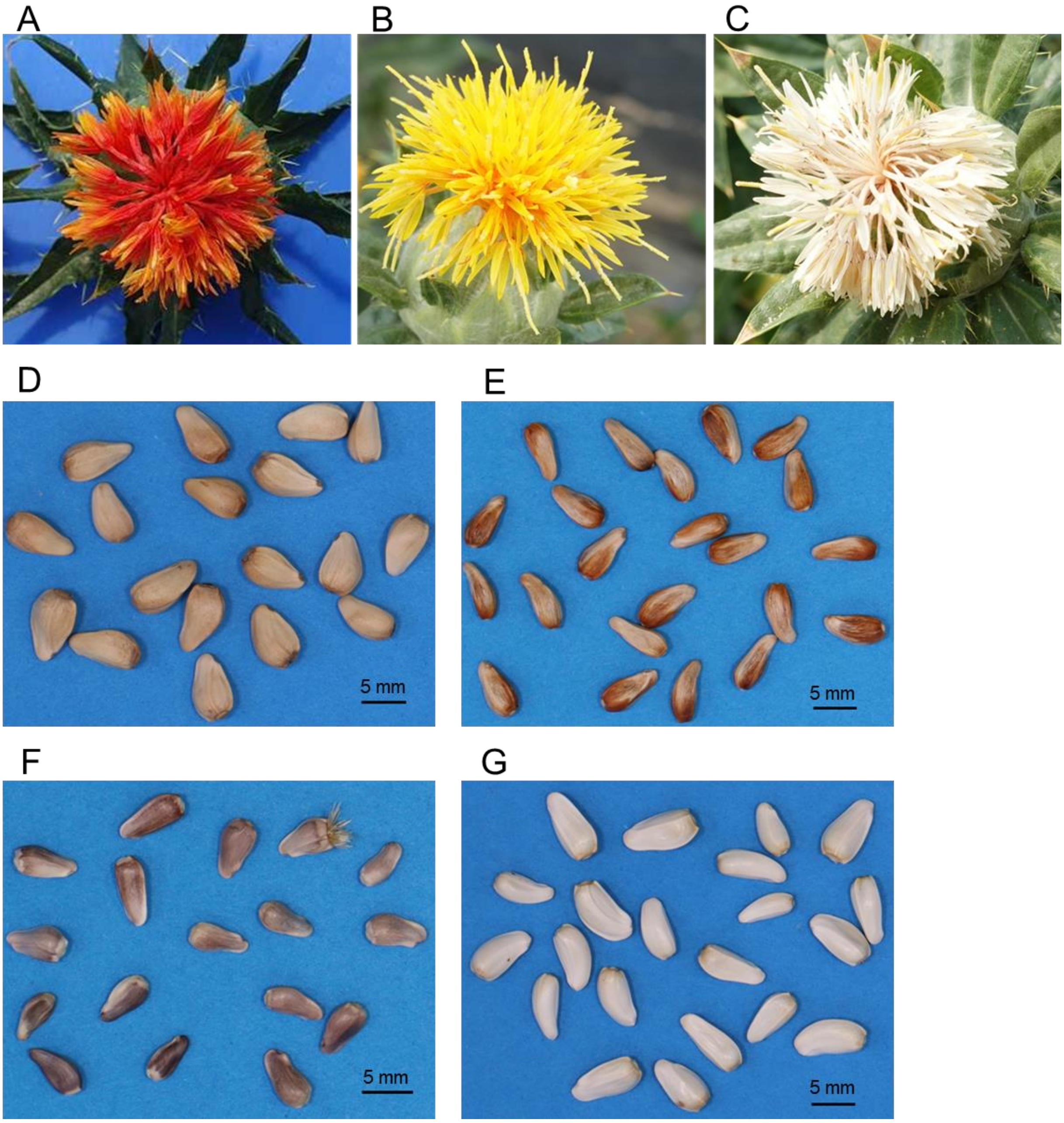

Figure S1. Flower and seed samples of safflower of different flower and seed coat colors. A: Red flower; B: Yellow flower; C: White flower; D: Light brown seed; E: Brown seed; F: Dark brown seed; G: White seed.

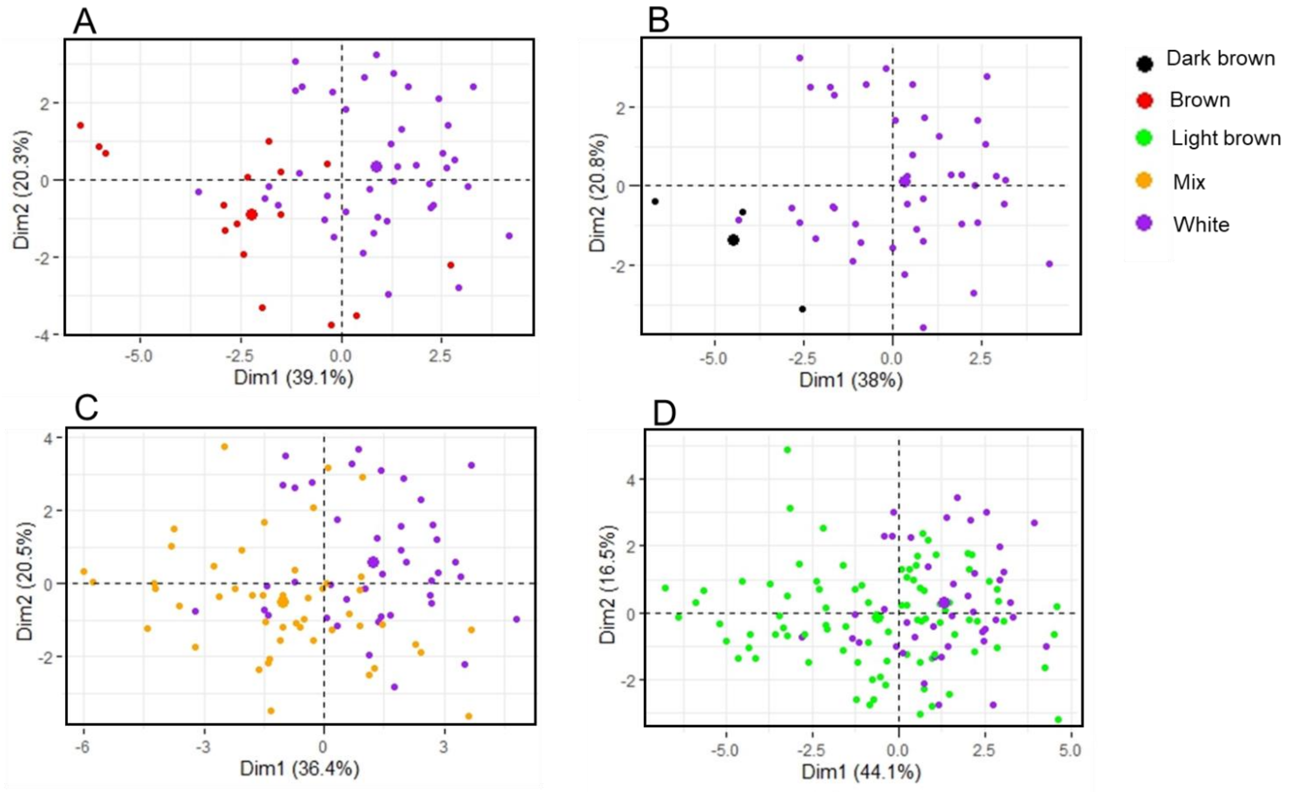

Figure S2. Principal component biplots of the total oil content, fatty acid profile, phytochemical contents, and antioxidant activities of the different seed coat color groups. (A) Loading plots of genotypes with white and brown seed coat colors; (B) Loading plots of genotypes with white and dark brown seed coat colors; (C) Loading plots of genotypes with white and mix seed coat colors; (D) Loading plots of genotypes with white and light brown seed coat colors.
